# West Nile virus infection in birds and mosquitoes, New York State, 2000.

**DOI:** 10.3201/eid0704.010415

**Published:** 2001

**Authors:** K A Bernard, J G Maffei, S A Jones, E B Kauffman, G Ebel, A P Dupuis, K A Ngo, D C Nicholas, D M Young, P Y Shi, V L Kulasekera, M Eidson, D J White, W B Stone, L D Kramer, NY State West Nile Virus Surveillance Team

**Affiliations:** Arbovirus Laboratories, New York State Department of Health, 5668 State Farm Road, Slingerlands, NY 12159, USA. bernard@wadsworth.org

## Abstract

West Nile (WN) virus was found throughout New York State in 2000, with the epicenter in New York City and surrounding counties. We tested 3,403 dead birds and 9,954 mosquito pools for WN virus during the transmission season. Sixty-three avian species, representing 30 families and 14 orders, tested positive for WN virus. The highest proportion of dead birds that tested positive for WN virus was in American Crows in the epicenter (67% positive, n=907). Eight mosquito species, representing four genera, were positive for WN virus. The minimum infection rate per 1,000 mosquitoes (MIR) was highest for Culex pipiens in the epicenter: 3.53 for the entire season and 7.49 for the peak week of August 13. Staten Island had the highest MIR (11.42 for Cx. pipiens), which was associated with the highest proportion of dead American Crows that tested positive for WN virus (92%, n=48) and the highest number of human cases (n=10).


### West Nile Virus Infection in Birds and Mosquitoes, New York State, 2000

**Kristen A. Bernard,* Joseph G. Maffei,* Susan A. Jones,* Elizabeth B. Kauffman,* Gregory D. Ebel,* Alan P. Dupuis II,* Kiet A. Ngo,* David C. Nicholas,* Donna M. Young,* Pei-Yong Shi,* Varuni L. Kulasekera,† Millicent Eidson,* Dennis J. White,* Ward B. Stone,‡ NY State West Nile Virus Surveillance Team,[1] and Laura D. Kramer***

*New York State Department of Health, Albany, New York, USA; †New York City Department of Health, New York, New York, USA; and ‡Department of Environmental Conservation, Albany, New York, USA

West Nile (WN) virus was found throughout New York State in 2000, with the epicenter in New York City and surrounding counties. We tested 3,403 dead birds and 9,954 mosquito pools for WN virus during the transmission season. Sixty-three avian species, representing 30 families and 14 orders, tested positive for WN virus. The highest proportion of dead birds that tested positive for WN virus was in American Crows in the epicenter (67% positive, n=907). Eight mosquito species, representing four genera, were positive for WN virus. The minimum infection rate per 1,000 mosquitoes (MIR) was highest for *Culex pipiens* in the epicenter: 3.53 for the entire season and 7.49 for the peak week of August 13. Staten Island had the highest MIR (11.42 for *Cx. pipiens*), which was associated with the highest proportion of dead American Crows that tested positive for WN virus (92%, n=48) and the highest number of human cases (n=10).

The emergence of West Nile (WN) virus in 1999 in four U.S. states (1) was followed by its spread to 12 states in 2000 (2). An enzootic cycle was established between birds and mosquitoes, and WN disease was observed in humans, horses, and birds in both years (2,3). Bird deaths due to WN virus are unusual outside North America, with the exception of deaths of geese in Israel (4) and pigeons in Egypt (5). In 1999 in North America, WN disease, characterized by meningoencephalitis and myocarditis, was observed in 14 species of wild and captive birds (6). WN virus has been detected in a number of mosquito genera in North America, including *Culex* and *Aedes* species (2,7). Vector competence has been confirmed experimentally for some North American species, including *Cx. pipiens*, *Ae. vexans*, and *Ae. japonicus* (8,9).

We have summarized surveillance data for WN virus in dead birds and mosquitoes for New York State in the 2000 transmission season. A quantitative and kinetic analysis of data within and outside the epicenter is shown for both the bird and mosquito samples. Vertebrate and invertebrate WN virus infections are compared for counties in the epicenter.

### Materials and Methods

### Bird and Mosquito Samples

Dead birds were collected and mosquitoes were trapped by local county health units and other agencies as part of the New York State WN virus surveillance effort. Dead birds were necropsied at the Wildlife Pathology Unit at the Department of Environmental Conservation. Kidney, brain, heart, liver, or spleen were harvested and stored at -70°C. Additional avian tissue samples sent to the National Wildlife Health Center were not included in this analysis because selection criteria and testing procedures differed. Mosquitoes were trapped, speciated, grouped into pools of 5 to 50, and stored at -70°C. For some pools, *Cx. pipiens* and *Cx. restuans* were not separated but were pooled together and denoted as *Cx. pipiens-restuans*. The *Aedes* genus is being reclassified into two genera, *Aedes* and *Ochlerotatus* (10), but is classified as *Aedes* in this manuscript. Avian tissue samples and mosquito pools were sent to the Arbovirus Laboratory at the Wadsworth Center for WN virus testing. The transmission season was defined as May 15 to October 31, 2000, with the first and last positive samples collected on May 22 and October 31, 2000, respectively. Two positive birds were found earlier in the year (a hawk on February 6 and a crow on April 1), but they were not followed by other positive specimens and did not therefore appear to represent the beginning of the mosquito-borne WN virus transmission season.

### WN Virus Testing

Samples were processed and WN virus assays were performed as described by Shi et al. (11). Briefly, RNA was extracted from bird tissue or pools of ≤50 mosquitoes, and real-time reverse transcription-polymerase chain reaction (RT-PCR) was performed (TaqMan, ABI Prism 7700 Sequence Detector, Applied Biosystems, Foster City, CA). Confirmatory tests included a second TaqMan primer-probe set, standard RT-PCR, virus isolation in cell culture, and

Address for correspondence: Kristen A. Bernard, Arbovirus Laboratories, Wadsworth Center, 5668 State Farm Road, Slingerlands, NY 12159; fax: 518-869-4530; e-mail: bernard@wadsworth.org

[1]P. Bryon Backenson, Ivan Gotham, Yoichiro Hagiwara, Geraldine S. Johnson, Gary Lukacik, Kathryn Schmit, and Amy L. Willsey, Division of Epidemiology, New York State Department of Health immunofluorescence assays (for avian tissues). A sample was confirmed as positive if at least two different test results were positive.

### Other Arbovirus Testing

Virus isolation was attempted for all *Aedes* species (with the exception of some *Ae. vexans* pools), all *Culiseta* species, and most WN virus-positive mosquito pools (with the exception of some *Cx. pipiens* and *Cx. pipiens-restuans* pools). Samples were inoculated onto monolayers of Vero or C6/36 cells. Viruses from positive cultures were typed by using serogroup-specific polyclonal antisera for bunyaviruses (California and Bunyamwera serogroups), flaviviruses, alphaviruses, and rhabdoviruses (Hart Park serogroup). California serogroup isolates were further characterized by sequence analysis.

### Definition of the Epicenter and Radial Regions

The epicenter of the New York State epizootic was defined as follows. The minimum infection rate (MIR) of each mosquito species was calculated for each county or New York City borough by the standard formula: (number of WN virus-positive mosquito pools/total number of mosquitoes tested) x 1000. The MIR was calculated only when at least 1,000 mosquitoes were tested per species per county or borough. When the MIR was at least 1.0 for any mosquito species in a region, the county or borough was included in the epicenter. In addition, any counties that bordered at least two other epicenter counties or boroughs were included in the definition. The epicenter included the five boroughs of New York City (the Bronx, Brooklyn, Manhattan, Queens, and Staten Island) and the four counties immediately east and north of New York City (Nassau, Suffolk, Rockland, and Westchester counties). For both 1999 and 2000, all human cases of WN virus in New York State were in one of these counties (2,7).

Counties outside the epicenter (the "non-epicenter") were divided into four radial regions, R1 to R4, with increasing distance from the epicenter (Figure 1). Radial regions were defined as follows: R1 = Putnam, Orange, Dutchess, Sullivan, and Ulster counties; R2 = Columbia, Delaware, Greene, Rensselaer, Montgomery, Albany, Otsego, Broome, Cortland, Schenectady, Schoharie, and Chenango counties; R3 = Fulton, Essex, Hamilton, Herkimer, Allegany, Lewis, Chemung, Madison, Cayuga, Schuyler, Yates, Washington, Warren, Tompkins, Tioga, Steuben, Onondaga, Seneca, Saratoga, Ontario, Oswego, and Oneida counties; and R4 = Monroe, Wyoming, Cattaraugus, Wayne, Chautauqua, Erie, Clinton, Genesee, Jefferson, Orleans, St. Lawrence, Niagara, Franklin, and Livingston counties.

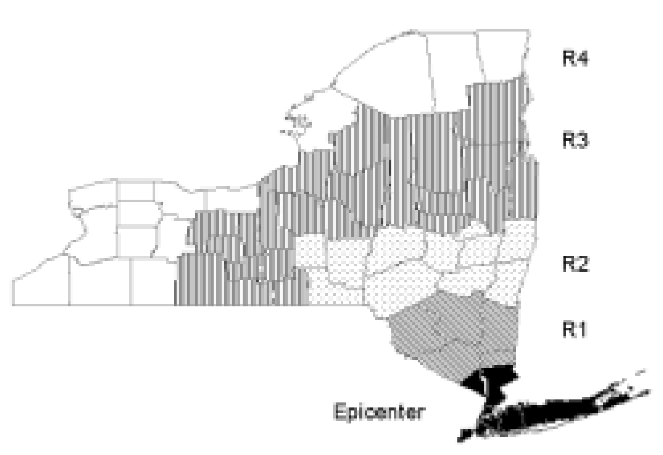

Figure 1. Map of New York State showing the epicenter and radial regions used for analysis. The non-epicenter was defined as R1, R2, R3, and R4. Counties included in the regions are defined in Materials and Methods.

### Results

During the 2000 transmission season, WN virus testing was performed on 3,403 dead birds, representing 153 species, 46 families, and 18 orders. The 1,201 WN virus-positive birds represented 63 species, 30 families, and 14 orders (Table 1). The percentage of WN virus-positive birds was 35% for all birds submitted for testing from throughout the state. Avian species that were >35% positive and for which at least 10 birds were tested included American Kestrel (57%, n=14), Cedar Waxwing (60%, n=10), Ovenbird (50%, n=18), American Crow (47%, n=1,687), Fish Crow (47%, n=45), and Red-tailed Hawk (43%, n=14). The discrepancies in number of birds tested make comparisons between species difficult.

The percentage of WN virus-positive birds was analyzed for the epicenter and non-epicenter regions. Data are included for avian species for which at least 10 birds were tested in one of the regions (Table 2). For all submitted birds, 51% and 23% WN virus-positive birds were found in the epicenter and non-epicenter regions, respectively. WN virus infection in dead birds was highest for American Crows (67%) in the epicenter. In the non-epicenter, WN virus infection for crows was lower, similar to infection in all birds in this region. High numbers of crows were tested in both regions, and the percentage positive differed by almost threefold.

WN virus infection in dead birds was examined over time for American Crows and all other birds in the epicenter and four radial regions in New York State (Figure 2). Comparison of American Crows in the five regions (Figure 2A) revealed the highest peak in the epicenter during September. In addition, the peak for American Crows in the epicenter was much broader than for the other four regions. For September, the peaks for American Crows in R1 and R2 were greater than those in the more distant regions, R3 and R4, suggesting a minor extension of the epicenter during this month. Comparison of all birds except American Crows in the five regions (Figure 2B) revealed little difference between the regions, even for the epicenter. These data support the hypothesis that the susceptibility to WN disease was greatest in crows in the epicenter.

We tested 9,954 mosquito pools with 317,668 mosquitoes, representing 28 species and eight genera (Table 3). Of eight positive species representing four genera, most positive pools were *Culex* species (n=341), compared with only 22 positive pools in the other three genera. All but five of the positive pools were collected in the epicenter. The MIR was calculated for each species in the epicenter for which at least 1,000 mosquitoes were tested (Table 3). In the epicenter, the MIR ranged from 0.47 to 3.55. The MIR of *Cx. pipiens* was the highest for an individual species. All the pure *Cx. restuans* pools were negative, and the MIR for *Cx. pipiens-restuans* was almost half that of the pure *Cx. pipiens* pools; therefore, the positive mosquitoes in the mixed pools of *Cx. pipiens-restuans*

Table 1. Birds positive for West Nile virus in New York State during the 2000 season[a]

| Order | Family | Common name | No. tested | % positive |
|---|---|---|---|---|
| Anseriformes | Anatidae | Domestic Goose | 2 | 50 |
| | | Canada Goose | 15 | 33 |
| | | Mute Swan | 3 | 33 |
| Apodiformes | Trochilidae | Ruby-throated Hummingbird | 5 | 20 |
| Caprimulgiformes | Caprimulgidae | Common Nighthawk | 2 | 50 |
| Charadriiformes | Charadriidae | Killdeer | 3 | 33 |
| | Laridae | Herring Gull | 9 | 33 |
| | | Ring-billed Gull | 66 | 32 |
| | | Greater Black-backed Gull | 7 | 29 |
| | Rynchopidae | Black Skimmer | 1 | 100 |
| | Scolopacidae | Ruddy Turnstone | 1 | 100 |
| Ciconiiformes | Ardeidae | Least Bittern | 1 | 100 |
| | | Green Heron | 3 | 33 |
| | | Great Blue Heron | 29 | 10 |
| Columbiformes | Columbidae | Mourning Dove | 83 | 19 |
| | | Rock Dove | 41 | 17 |
| Coraciiformes | Alcedinidae | Belted Kingfisher | 6 | 33 |
| Falconiformes | Accipitridae | Red-tailed Hawk | 14 | 43 |
| | | Sharp-shinned Hawk | 17 | 35 |
| | | Cooper's Hawk | 30 | 30 |
| | | Broad-winged Hawk | 7 | 14 |
| | Falconidae | Merlin | 5 | 100 |
| | | American Kestrel | 14 | 57 |
| Galliformes | Meleagrididae | Domestic Turkey | 1 | 100 |
| | | Eastern Wild Turkey | 3 | 67 |
| | Phasianidae | Peacock | 8 | 25 |
| | | Ring-necked Pheasant | 16 | 25 |
| | Tetraonidae | Chicken | 14 | 29 |
| | | Ruffed Grouse | 131 | 21 |
| Gruiformes | Rallidae | Virginia Rail | 2 | 50 |
| Passeriformes | Bombycillidae | Cedar Waxwing | 10 | 60 |
| | Corvidae | Fish Crow | 45 | 47 |
| | | American Crow | 1,687 | 47 |
| | | Blue Jay | 500 | 29 |
| | Fringillidae | Zebra Finch | 1 | 100 |
| | | Song Sparrow | 5 | 60 |
| | | American Goldfinch | 4 | 50 |
| | | House Finch | 8 | 38 |
| | | Cardinal | 3 | 33 |
| | Icteridae | Red-winged Blackbird | 6 | 17 |
| | | Common Grackle | 53 | 13 |
| | Mimidae | Gray Catbird | 22 | 23 |
| | | Northern Mockingbird | 10 | 20 |
| | Parulidae | Black-throated Blue Warbler | 1 | 100 |
| | | Canada Warbler | 1 | 100 |
| | | Warbler | 1 | 100 |
| | | Yellow-rumped Warbler | 1 | 100 |
| | | Ovenbird | 18 | 50 |
| | Ploceidae | House Sparrow | 127 | 13 |
| | Sturnidae | European Starling | 23 | 17 |
| | Turdidae | Veery | 3 | 33 |
| | | Eastern Bluebird | 4 | 25 |
| | | American Robin | 74 | 22 |
| | | Wood Thrush | 5 | 20 |
| | Tyrannidae | Eastern Phoebe | 2 | 50 |
| Pelecaniformes | Phalacrocoracidae | Cormorant | 2 | 100 |
| | | Double Crested Cormorant | 2 | 50 |
| Psittaciformes | Cacatuidae | Cockatoo | 1 | 100 |
| | | Cockatiel | 5 | 60 |
| | Psittacidae | Macaw | 1 | 100 |
| | | Parakeet | 9 | 22 |
| Strigiformes | Strigidae | Snowy Owl | 2 | 100 |
| | | Great Horned Owl | 16 | 19 |

[a]Season defined as May 15, 2000, through October 31, 2000.

Table 2. Summary of birds tested for West Nile virus in New York during the 2000 season[a]

| | Epicenter | | Non-epicenter | |
|---|---|---|---|---|
| Common name | No. tested | % WN virus positive | No. tested | % WN virus positive |
| American Crow | 907 | 67 | 780 | 23 |
| Fish Crow | 31 | 55 | 14 | 29 |
| Blue Jay | 191 | 40 | 309 | 23 |
| Cooper's Hawk | 11 | 27 | 19 | 32 |
| Sharp-shinned Hawk | <10 | NA[b] | 14 | 36 |
| American Robin | 11 | 9 | 59 | 22 |
| House Sparrow | 107 | 8 | 20 | 40 |
| European Starling | 15 | 7 | <10 | NA |
| Common Grackle | 27 | 7 | 26 | 19 |
| Gray Catbird | <10 | NA | 16 | 25 |
| Ovenbird | <10 | NA | 12 | 75 |
| Common Yellow Throat | 19 | 0 | <10 | NA |
| Mallard | <10 | NA | 12 | 0 |
| Ring-billed Gull | <10 | NA | 66 | 32 |
| Great Blue Heron | <10 | NA | 28 | 7 |
| Rock Dove | 16 | 0 | 25 | 28 |
| Mourning Dove | <10 | NA | 77 | 19 |
| Ring-necked Pheasant | <10 | NA | 15 | 27 |
| Chicken | <10 | NA | 10 | 30 |
| Ruffed Grouse | <10 | NA | 130 | 21 |
| Great Horned Owl | <10 | NA | 15 | 20 |
| Total[c] | 1,502 | 51 | 1,901 | 23 |

[a]Season defined as May 15, 2000, through October 31, 2000. Bird species were included only if at least 10 birds were tested for one of the regions throughout the season.
[b]NA = not applicable because of the low number of birds tested.
[c]All birds tested in each region throughout the season.

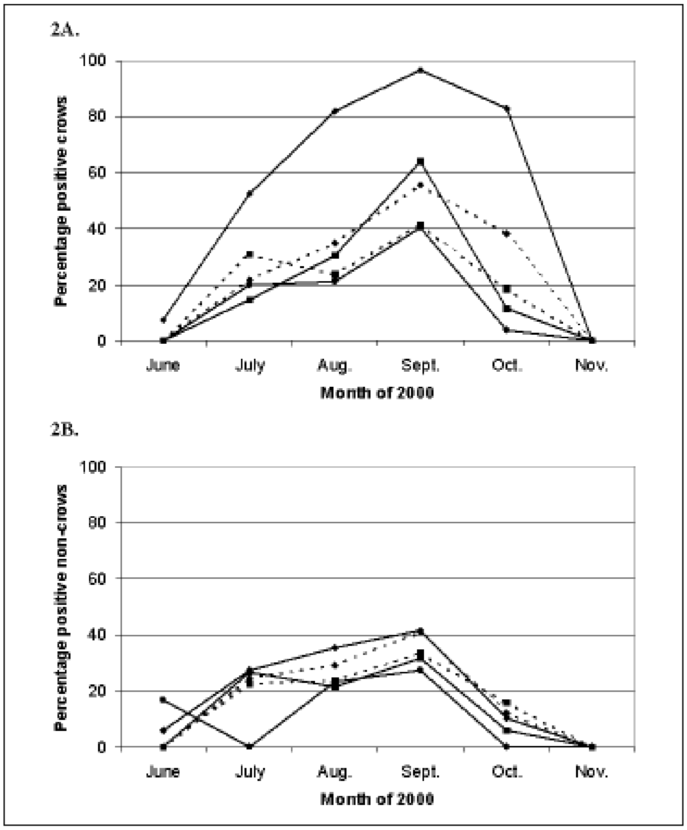

Figure 2. Percentage of West Nile virus-positive birds in different regions of New York State over time. Percentage of positive American Crows (2A) or birds excluding American Crows (2B) in the epicenter and radial regions with increasing distance from the epicenter, R1 to R4 (Figure 1). The epicenter (◆ solid line), R1(◆ dashed line), R2 (▪ solid line), R3 (▪ dashed line), and R4 (• solid line).

were most likely *Cx. pipiens*. The MIRs for *Cx. pipiens* and *Cx. pipiens-restuans* were compared with the percentage of positive American Crows in the epicenter for each week of the transmission season (Figure 3). The MIRs for *Cx. pipiens* and *Cx. pipiens-restuans* peaked 3 and 2 weeks, respectively, before the peak for positive crows. These data support the hypothesis that *Cx. pipiens* is an important enzootic vector of WN virus in New York.

The epicenter was examined as individual boroughs and counties to compare vertebrate and invertebrate WN virus infections (Table 4). The percentage of positive American Crows was calculated, and human and equine cases were noted for each county over the entire season. The MIRs of mosquitoes from each county were calculated for species with at least 1000 mosquitoes tested. Six mosquito species or groups met this criterion. The highest number of vertebrates infected with WN virus was found in Staten Island and was associated with the highest mosquito MIRs. This borough had measurable MIRs for five mosquito groups: *Cx. pipiens*, *Cx.* species, *Cx. pipiens-restuans*, *Cx. salinarius,* and *Ae. vexans.*

Virus isolation was performed on mosquito samples by cell culture. WN virus was isolated from 110 samples, including *Cx. pipiens-restuans* (n=60), *Cx. pipiens* (n=25), *Cx. salinarius* (n=13), *Culex* species (n=9), *Ae. triseriatus* (n=1), *Ae. vexans* (n=1), and *Psorophora ferox* (n=1). No virus was isolated from any of the WN virus RNA-positive pools of *Ae. japonicus*, *Ae. cantator*, and *An. punctipennis*. Pools that were negative on Vero cell culture were passaged repeatedly in *Aedes albopictus* (C6/36) cells; these further attempts to isolate virus were unsuccessful. Other viruses isolated were trivittatus virus from *Ae. trivittatus* (n=4) and *Ae. triseriatus* (n=1), Cache Valley virus from *Ae. trivittatus* (n=2) and *Ae. triseriatus* (n=2), and Flanders virus from *Cx. pipiens-restuans* (n=7), *Cx. pipiens* (n=2), and *Cs. melanura* (n=11). Eastern equine encephalitis virus and California group viruses other than trivittatus were not isolated from any of the *Culiseta* or *Aedes* pools.

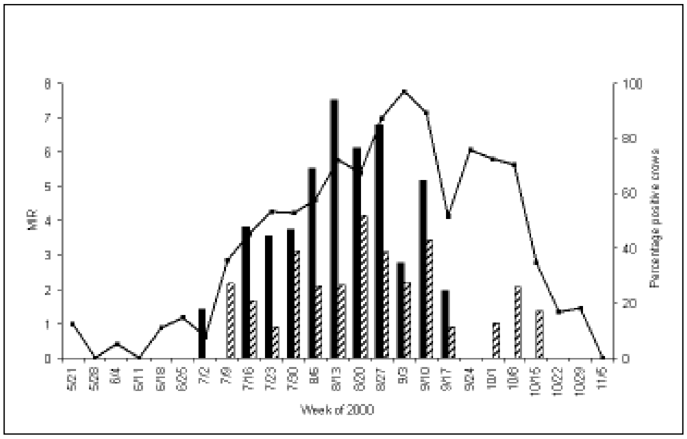

Figure 3. Weekly minimal infection rate per 1,000 mosquitoes (MIR) and percentage of crows positive for West Nile virus in the epicenter. Solid bars designate *Culex pipiens*. Hatched bars designate *Cx. pipiens-restuans*. Solid line designates percentage of positive crows.

Table 3. Summary of mosquitoes tested for West Nile virus in New York State in 2000

| Mosquito species | Epicenter: Total mosquitoes | Epicenter: Total pools | Epicenter: Total positive pools | Epicenter: MIR[a] | Non-epicenter: Total mosquitoes | Non-epicenter: Total pools | Non-epicenter: Total positive pools |
|---|---|---|---|---|---|---|---|
| *Aedes abserratus-punctor* | NC[b] | NC | NC | NC | 214 | 9 | 0 |
| *Ae. canadensis* | 8,018 | 167 | 0 | 0 | 8,269 | 145 | 0 |
| *Ae. cantator* | 1,615 | 65 | 1 | 0.62 | 993 | 22 | 0 |
| *Ae. cinereus* | 340 | 15 | 0 | NA[c] | 247 | 10 | 0 |
| *Ae. communis* | NC | NC | NC | NC | 335 | 12 | 0 |
| *Ae. communis* group | NC | NC | NC | NC | 235 | 8 | 0 |
| *Ae. intrudens* | 21 | 1 | 0 | NA | NC | NC | NC |
| *Ae. japonicus* | 3,342 | 257 | 2 | 0.60 | 3,871 | 271 | 3 |
| *Ae. sollicitans* | 5,003 | 131 | 0 | NA | 1 | 1 | 0 |
| *Ae. stimulans* | NC | NC | NC | NC | 42 | 1 | 0 |
| *Ae. stimulans* group | 153 | 3 | 0 | NA | 540 | 17 | 0 |
| *Ae. taeniorhynchus* | 395 | 11 | 0 | NA | 11 | 1 | 0 |
| *Ae. trichuris* | NC | NC | NC | NC | 23 | 2 | 0 |
| *Ae. triseriatus* | 2,956 | 203 | 3 | 1.01 | 6,335 | 206 | 0 |
| *Ae. trivittatus* | 4,289 | 184 | 0 | 0 | 2,453 | 79 | 0 |
| *Ae. vexans* | 21,486 | 761 | 10 | 0.47 | 13,490 | 422 | 0 |
| *Aedes* species | 1,340 | 72 | 1 | 0.75 | NC | NC | NC |
| *Anopheles crucians* | 13 | 2 | 0 | NA | NC | NC | NC |
| *An. punctipennis* | 165 | 37 | 1 | NA | 291 | 17 | 0 |
| *An. quadrimaculatus* | 66 | 18 | 0 | NA | 59 | 3 | 0 |
| *An. walkeri* | NC | NC | NC | NC | 833 | 22 | 0 |
| *Anopheles* species | 16 | 2 | 0 | NA | NC | NC | NC |
| *Coquillettidia perturbans* | 8,167 | 157 | 0 | 0 | 10,874 | 206 | 0 |
| *Culiseta melanura* | 8,189 | 211 | 0 | 0 | 6,281 | 178 | 0 |
| *Cs. morsitans* | NC | NC | NC | NC | 1,821 | 85 | 0 |
| *Culiseta* species | 98 | 3 | 0 | NA | NC | NC | NC |
| *Culex pipiens* | 22,120 | 831 | 78 | 3.53 | 8,698 | 288 | 1 |
| *Cx. pipiens-restuans*[d] | 114,517 | 3,208 | 211 | 1.84 | 16,228 | 537 | 1 |
| *Cx. restuans* | 3,403 | 190 | 0 | 0 | 794 | 48 | 0 |
| *Cx. salinarius* | 19,541 | 483 | 31 | 1.59 | 704 | 17 | 0 |
| *Cx. territans* | 76 | 15 | 0 | NA | NC | NC | NC |
| *Culex* species | 5,358 | 200 | 19 | 3.55 | 1,108 | 32 | 0 |
| *Orthopodomyia alba* | NC | NC | NC | NC | 101 | 3 | 0 |
| *Psorophora ferox* | 63 | 10 | 1 | NA | 162 | 6 | 0 |
| *Uranotaenia sapphirina* | 208 | 19 | 0 | NA | 419 | 18 | 0 |
| Unidentified | 1,173 | 26 | 0 | 0 | 105 | 6 | 0 |
| TOTAL | 232,131 | 7,282 | 358 | | 85,537 | 2,672 | 5 |

[a] MIR = minimal infection rate per 1,000 mosquitoes. MIR calculated as (number of WN virus-positive pools by RT-PCR/total mosquitoes tested) x 1,000. MIR was calculated only if a minimum of 1,000 mosquitoes was tested from a defined region throughout the season. None of the counties outside the epicenter met this criterion.
[b]NC = not collected.
[c]NA = not applicable because <1,000 mosquitoes were collected.
[d]*Cx. pipiens* and *Cx. restuans* were not separated and were pooled together.

Table 4. Comparison of infection in vertebrates and minimal infection rate in mosquitoes for WN virus in the epicenter of the New York epizootic of 2000

| Borough or county | % positive crows (n)[b] | No. human cases[c] | No. equine cases[d] | MIR[a]: *Culex pipiens* | MIR[a]: *Cx. pipiens-restuans*[e] | MIR[a]: *Culex* species | MIR[a]: *Cx. salinarius* | MIR[a]: *Aedes vexans* | MIR[a]: *Ae. japonicus* |
|---|---|---|---|---|---|---|---|---|---|
| Staten Island | 92% (48) | 10 | 1 | 11.42 | 9.9 | 6.92 | 2.61 | 0.79 | NC[f] |
| Brooklyn | 73% (48) | 2 | 0 | 3.12 | 1.42 | NA[g] | 0.67 | NA | NC |
| Manhattan | 85% (34) | 1 | 0 | 2.91 | 3.86 | NA | NA | NA | NC |
| Queens | 64% (25) | 1 | 0 | 0.16 | 0.24 | 0 | 0.2 | NA | NA |
| Suffolk | 70% (188) | 0 | 8 | NC | 2.74 | NC | NC | 0.40 | NA |
| Bronx | 44% (9) | 0 | 2 | 2.38 | NA | NA | 0.87 | 0 | NC |
| Rockland | 76% (280) | 0 | 0 | NA | 1.98 | NA | NA | 0.44 | NA |
| Westchester | 44% (128) | 0 | 0 | 0.51 | 0.73 | NC | NC | 0 | 0.43 |
| Nassau | 56% (147) | 0 | 4 | NC | 0.28 | NC | NC | 0.45 | NC |

[a]MIR = minimal infection rate per 1,000 mosquitoes. Mosquito species were included only if a minimum of 1,000 total mosquitoes was collected throughout the season for the county. MIR was calculated as (number of WN virus-positive pools by RT-PCR/total mosquitoes tested) x 1,000.
[b]Percentage WN virus-positive crows throughout the transmission season with total number of crows tested in parentheses.
[c]Human cases reported by the Centers for Disease Control and Prevention (2).
[d]Equine cases reported by S. Trock (personal communication).
[e]*Cx. pipiens* and *Cx. restuans* were not separated and were pooled together.
[f]NC = not collected.
[g]NA = not applicable because <1,000 mosquitoes were collected.

### Conclusion

In the 2000 transmission season in New York State, we found 63 bird species infected with WN virus, compared with 14 species in 1999 (6). The percentage of WN virus-positive birds was higher in the epicenter than outside it. This high percentage almost entirely reflects infected crows in the epicenter; no increase in WN virus infection was noted in birds other than crows. In contrast, high WN virus infection of dead crows was not observed outside the epicenter, where the percentage of WN virus positivity was similar in crows and other birds over the entire season. High numbers of dead crows were also observed in 1999 (3,6). The cause of the increased sensitivity of crows to WN disease or infection is unknown, but may be due to virus-host interactions, mosquito-bird interactions, mosquito feeding preferences, crow population density, or crow behavior. The presence of WN virus in dead birds does not indicate a definitive diagnosis of WN virus as the cause of death. Many of the birds did not show gross pathologic lesions of WN disease (12). In addition, the rate of WN virus-positive birds in our surveillance samples is not equivalent to prevalence of infection, since we are sampling only dead birds.

The analysis of percentage positive birds over time revealed differences between various regions in New York State. The percentage of WN virus-positive crows was highest in the epicenter compared with other regions of New York State throughout the season, supporting the importance of crows as indicators in the epizootic. The percentage of WN virus-positive crows was higher than that for all other birds early in the season only in the epicenter. Outside the epicenter, the percentages of WN virus-positive crows and all other birds were similar until the peak month of September. At this time, the two radial regions closest to the epicenter showed higher infection in crows than in other birds, suggesting that the intense level of viral activity may have spread beyond the epicenter. An explanation for this apparent spread may be increased movement of crows during the fall. The surveillance data on avian deaths have implications for future surveillance activities. The similar percentages of positive crows and other birds outside the epicenter indicate the importance of testing all birds, not only crows, outside the epicenter.

Sampling errors are likely with the avian surveillance samples. The samples were from dead birds submitted to the Wildlife Pathology Unit, which relied on the cooperation of the general public, individual county health departments, and other agencies; therefore, surveillance samples do not represent a random sampling of dead birds throughout the state. The size and degree of urbanization of various bird species may have resulted in differences in submission. For example, large urban-dwelling species, such as crows, were more likely to have been submitted than small rural dwellers. In addition, more birds were likely sampled from areas with human cases, greater media coverage, and higher human population. The similarity in WN virus infection in birds other than crows from different regions suggests that the impact of sampling bias was not significant. Additionally, specimens submitted for testing to the Wadsworth Center from the Wildlife Pathology Unit represent a sample of those submitted by the public. Sampling at this level may introduce further bias into our surveillance sample. The similarity in WN virus infection in birds other than crows outside the epicenter, however, suggests that such bias was minimal.

The highest MIR for mosquitoes in the epicenter was for *Cx. pipiens*. Positive pools of *Cx. pipiens* also were identified in 1999 in New York (13). *Cx. pipiens-pipiens* mosquitoes feed almost exclusively on birds (14); thus, they are likely an important enzootic vector in the bird-mosquito cycle in North America. Other *Cx.* species have been implicated as enzootic vectors worldwide (15). High MIRs of *Cx. pipiens* and *Cx. salinarius* were associated with human and equine WN virus cases and high infection rates of crows in counties in the epicenter. *Cx. salinarius* feeds on both birds and mammals (16); therefore, it is a likely candidate as a "bridge vector," transmitting the virus from bird to mammal. *Cx. salinarius* has been proposed as a bridge vector for Eastern equine encephalitis virus (17). All MIR data were calculated by using RT-PCR-positive pools and therefore cannot be directly compared with MIRs calculated by using infectious virus-positive pools. No virus was isolated from five RNA-positive pools of *Ae. japonicus*, even after six serial passages through C6/36 cells. Much attention has been focused on this species because it has been reported to be a highly competent laboratory vector of WN virus (9), but current field data do not support this experimental observation. Different populations of *Ae. japonicus* have been described in the eastern United States (18), and differences in vector competence between the populations may explain the discrepancy between the field and experimental data.

The possibility of sampling error also exists for the mosquito surveillance samples. Individual counties collected mosquitoes in different numbers and set traps by different criteria. In addition, some counties used mosquito adulticides or larvicides during the season. These sources of bias are unlikely to have been uniformly introduced, and their impact on our analyses is unclear.

The results from the 2000 surveillance season for WN virus leave a number of unanswered questions. Many avian species can become infected with WN virus, but the prevalence of infection for each species is unknown without systematic serosurveys of the wild bird population. It is also unknown which birds have a high enough viremia for efficient transmission to the vector. The apparent mortality rate caused by WN virus is higher for crows than for other birds, but laboratory experiments are required to determine WN virus mortality rates and the pathogenic mechanisms in crows and other avian species. In addition to crows, many other birds, such as raptors and other corvids, also showed significant pathology. Some nonmigrating species (e.g., ruffed grouse) have potential use as an indicator species for WN virus infection. Vector competence, blood meal identification, and transovarial transmission studies for the potential mosquito vectors are also important research areas.

### Acknowledgments

We thank the WN virus surveillance team, especially the New York City Department of Health and the local county health units, who collected the surveillance specimens. We also thank H. Taber for helpful discussions.

Support was provided by the Wadsworth Center and the Division of Epidemiology, New York State Department of Health, the Department of Environmental Conservation, and the Centers for Disease Control and Prevention.

Dr. Bernard is assistant director of the Arbovirus Laboratory at the Wadsworth Center. Her major research interests are viral pathogenesis and the maintenance of arboviruses in temperate climates.

### References

1. Lanciotti RS, Roehrig JT, Deubel V, Smith J, Parker M, Steele K, et al. Origin of the West Nile virus responsible for an outbreak of encephalitis in the northeastern United States. Science 1999;286:2333-7.
2. Centers for Disease Control and Prevention. Update: West Nile Virus activity—Eastern United States, 2000. MMWR Morb Mortal Wkly Rep 2000;49:1044-7.
3. Centers for Disease Control and Prevention. Outbreak of West Nile-like viral encephalitis—New York, 1999. MMWR Morb Mortal Wkly Rep 1999;48:845-9.
4. Office of Internationale des Epizooties. West Nile fever in Israel in geese. Disease Information 1999;12:166.
5. Work TH, Hurlbut HS, Taylor RM. Isolation of West Nile virus from hooded crow and rock pigeon in the Nile Delta. Proc Soc Exp Biol Med 1953;84:719-22.
6. Steele KE, Linn MJ, Schoepp RJ, Komar N, Geisbert TW, Manduca RM, et al. Pathology of fatal West Nile virus infections in native and exotic birds during the 1999 outbreak in New York City, New York. Vet Pathol 2000;37:208-24.
7. Centers for Disease Control and Prevention. Update: West Nile virus encephalitis—New York, 1999. MMWR Morb Mortal Wkly Rep 1999;48:944-55.
8. Turell MJ, O'Guinn M, Oliver J. Potential for New York mosquitoes to transmit West Nile virus. Am J Trop Med Hyg 2000;62:413-4.
9. O'Guinn ML, Dohm DJ, Jones JW, Turell MJ. Potential mosquito vectors of West Nile virus in the United States. Am J Trop Med Hyg 2001;62:286.
10. Reinert JF. New classification for the composite genus *Aedes* (Diptera: Culicidae: Aedini), elevation of subgenus *Ochlerotatus* to generic rank, reclassification of other subgenera, and notes on certain subgenera and species. J Am Mosq Control Assoc 2000;16:175-88.
11. Shi P-Y, Kauffman EB, Ren P, Felton A, Tai JH, Dupuis II AP, et al. High throughput detection of West Nile virus RNA. J Clin Microbiol 2001;39:1264-71.
12. Eidson M, Kramer LD, Stone W, Hagiwara Y, Schmit K, New York State West Nile Virus Avian Surveillance Team. Dead bird surveillance as an early warning system for West Nile virus. Emerg Infect Dis 2001;7:631-5.
13. Centers for Disease Control and Prevention. Update: West Nile-like viral encephalitis—New York, 1999. MMWR Morb Mortal Wkly Rep 1999;48:890-2.
14. Spielman A. Population structure in the *Culex pipiens* complex of mosquitos. Bull World Health Organ 1967;37:271-6.
15. Hubalek Z, Halouzka J. West Nile fever—a reemerging mosquito-borne viral disease in Europe. Emerg Infect Dis 1999;5:643-50.
16. Crans WJ. Continued host preference studies with New Jersey mosquitoes, 1963. In: Proceedings of the Annual Meeting of the New Jersey Mosquito Extermination Association 1964;51:50-8.
17. Vaidyanathan R, Edman JD, Cooper LA, Scott TW. Vector competence of mosquitoes (Diptera: Culicidae) from Massachusetts for a sympatric isolate of eastern equine encephalomyelitis virus. J Med Entomol 1997;34:346-52.
18. Fonseca DM, Campbell SR, Crans WJ, Mogi M, Miyagi I, Toma T, et al. *Aedes* (Finlaya) *japonicus* (Diptera: Culicidae), a newly recognized mosquito in the United States: Analyses of genetic variation in the United States and putative source populations. J Med Entomol 2001;38:135-46.
